# Complement role in kidney disease: a comprehensive review and therapeutic innovations

**DOI:** 10.1186/s12882-026-04880-7

**Published:** 2026-03-06

**Authors:** Beatriz Cortez Ferreira, Gabriela Matos Silva, João Venda, Nuno Afonso, Rita Leal, Sofia Cerqueira, Ana Galvão, Helena Sá

**Affiliations:** 1 Nephrology Department, Unidade Local de Saúde de Coimbra, Coimbra, Portugal; 2 Faculty of Medicine, University of Coimbra, Coimbra, Portugal

**Keywords:** Complement activation, Immune-mediated kidney diseases, Targeted therapy, Complement inhibitors, Glomerular diseases, Kidney transplantation

## Abstract

The complement system is a vital component of innate immunity, known for its role in pathogen defense. It has been increasingly recognized as a mediator of homeostatic clearance, tissue repair, and immune-metabolic crosstalk. In the kidney, unique anatomical and hemodynamic features predispose to aberrant complement activation, rendering glomerular structures particularly vulnerable. Complement dysregulation is implicated in a broad spectrum of kidney diseases – ranging from primary complement-driven disorders (atypical hemolytic uremic syndrome, C3 glomerulopathy, and emergent C4 glomerulopathy) to secondary complement disorders, with special emphasis on classical immune complex diseases, podocytopathies, and kidney transplantation. Within glomeruli, complement activation triggers non-lytic cellular activation (including calcium influx, kinase signaling, cytokine release, and cytoskeletal remodeling), inflammation, cytotoxic injury, and maladaptive remodeling via effectors such as C3a, C5a, and the membrane attack complex (C5b-9). Genetic and autoantibody–mediated defects in complement regulators amplify this risk. Recent advances in complement-targeted therapies—including inhibitors of C3, C5, factor B, and MASP-2—are entering glomerular disease trials, with early evidence of efficacy in proteinuria reduction and slowing of glomerular injury. Nevertheless, key challenges remain: balancing infection risk, defining appropriate durations, selecting responsive patient populations based on complement pathway profiling, and achieving cost-effectiveness. Looking ahead, integration of genetic, proteomic, and histopathologic biomarkers will be essential to implement a precision-medicine approach. Complement remains a frontier in glomerular disease research, bridging fundamental immunology to translational nephrology and offering new pathways to modify disease progression.

## Clinical trial number

Not applicable.

## Background

**Fig. 1:**
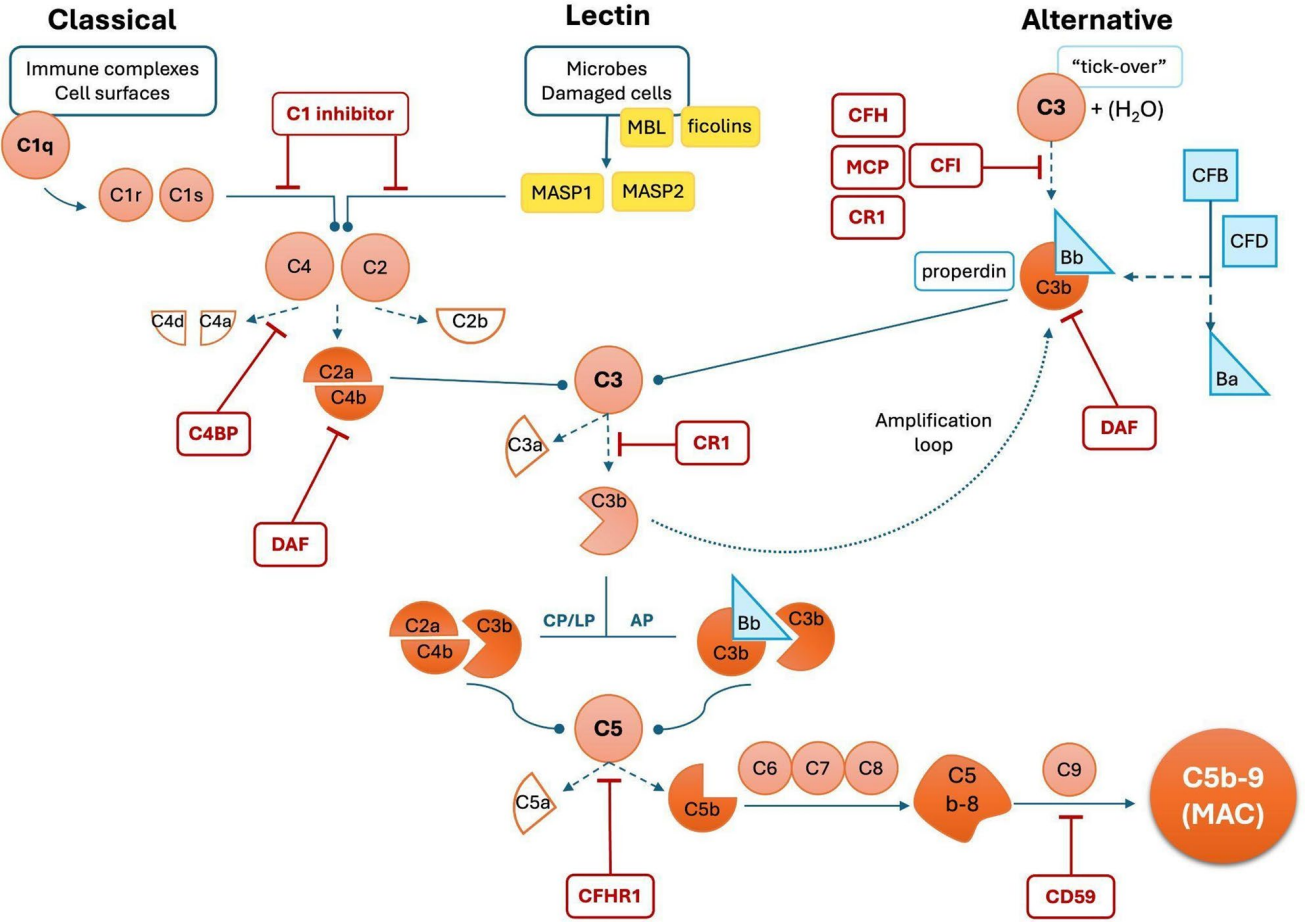
Complement pathway. C: complement component; CD59: protectin; CFB: complement factor B; CFD: complement factor D; CFH: complement factor H; CFHR1: complement factor-H related protein 1; CFI: complement factor I; CR1: complement receptor 1; C4BP: C4 binding protein; DAF: decay accelerator factor; MAC: membrane attack complex; MASP: mannan-binding lectin serine protease; MBL: mannose-binding lectin; MCP: membrane cofactor protein

The complement system is a key component of innate immunity, mediating pathogen defense, immune surveillance, apoptotic clearance, and tissue homeostasis [1, 2]. Its functions extend into cross-talk with coagulation, adaptive immunity, and repair pathways. The complement cascade comprises more than 30 plasma and membrane-anchored proteins, which can be activated via the classical, lectin, and alternative pathways, all converging at the central hub of C3 (Fig. 1) [1–4]. Activation involves tightly regulated proteolytic cascades in which inactive zymogens are sequentially cleaved into active serine proteases, generating downstream effectors, including the anaphylatoxins (C3a, C5a) and the membrane attack complex (C5b-9; MAC), which mediate inflammation, cell activation, and cytolysis [2, 3].

While complement is indispensable for host defense, its potent effector system also harbors the risk of self-injury. The kidney, particularly the glomerulus, has a high proclivity to complement-mediated damage [1, 3–5]. Several unique features underlie this vulnerability: (i) high glomerular hydrostatic pressures; (ii) filtration of plasma across fenestrated endothelium increases exposure of large complement proteins to glomerular structures; (iii) localized paucity of intrinsic complement regulators in the glomerular basement membrane may impair local protection [4, 5]. 

Dysregulated complement activation is now implicated across a spectrum of glomerular diseases, either as a primary driver, or as a secondary effector in immune complex–driven and non–immune glomerular lesions [1, 3, 4]. Within the glomeruli, complement induces cell stress, inflammation, structural injury, and maladaptive remodeling [3]. 

In this review, we detail the pathways of complement activation in the kidney, illustrate the spectrum of complement-mediated glomerulopathies, and describe the emerging complement-targeted therapies, highlighting current challenges and future directions.

## Complement pathways and renal involvement

Complement activation follows a simple and logical sequence of surface recognition and opsonization of the target cell, self-amplification, generation of effector molecules, and induction of immune signaling [2]. 

### Classical pathway (CP)

The classical pathway is typically activated when the recognition molecule C1q binds to antigen-antibodies complexes, usually IgM or IgG. C1r/C1s activation cleaves C4 and C2, forming C4b2a. This C3 convertase complex (C4b2a) catalyzes the cleavage of C3 into C3a and C3b [6]. 

### Lectin pathway (LP)

The lectin pathway becomes activated when soluble pattern recognition molecules (e.g. mannose-binding lectin (MBL) or ficolins) bind to specific sugar structures found on the surface of microbes or damaged cells. Once bound, these molecules recruit and activate mannan-binding lectin serine protease (MASP)-1 and MASP-2, leading to the cleavage of C4 and C2, and formation of the C3 convertase complex [6]. The LP appears to be particularly important in detecting injury or altered cell surfaces in the kidney [6]. 

### Alternative pathway (AP)

The alternative pathway is constitutively active at low “tick-over” rate, in which spontaneous hydrolysis of native C3 produces a fluid-phase C3(H₂O) that can form a convertase with factor B (CFB) and factor D (CFD) [6]. Once C3b is deposited on a nearby surface, it can bind CFB, which is cleaved by CFD to generate the surface-bound alternative pathway C3 convertase, C3bBb, which is stabilized by properdin [7, 8]. The AP acts as an amplification loop for the other two pathways, enhancing the deposition of C3 fragments [6, 7]. In glomeruli, local conditions (e.g., low regulator expression or high complement flux) may favor AP overactivity [4, 8]. 

### Terminal pathway (TP)

#### TP and C5b-9

The terminal pathway of complement is responsible for its main effector functions. Once C3b accumulates on surfaces, cleavage of C5 by C5 convertase yields C5a (a potent anaphylatoxin and chemotactic mediator) and C5b, which triggers assembly of C5b-9 [6]. In bacteria and erythrocytes, this leads to cell lysis; whereas in nucleated host cells, it induces intracellular calcium influx and activation of downstream signaling pathways, leading to intracellular signaling, metabolic stress, and cytokine release [2, 4]. 

The biological effects of sub-lytic MAC are cell-type dependent. In mesangial cells, C5b-9 promotes proliferation and extracellular matrix production, contributing to mesangial expansion; in glomerular endothelial cells, it induces pro-inflammatory and pro-thrombotic signaling; and in podocytes, it disrupts actin cytoskeletal organization and slit diaphragm integrity, promoting proteinuria [4, 9–11]. 

In addition to membrane-bound C5b-9, TP activation generates soluble C5b-9 (sC5b-9), a non-lytic complex formed in the fluid phase when vitronectin and clusterin prevent membrane insertion of C5b-7. This process represents a physiological regulatory mechanism limiting terminal pathway–mediated cytotoxicity rather than a passive byproduct of activation [2, 6].

### Anaphylatoxins

The anaphylatoxins C3a and C5a exert their biological effects through distinct G-protein–coupled receptors (R), C3aR and C5aR1, which are expressed in renal parenchymal cells. In the kidney, C3aR and C5aR1 signaling promotes inflammatory, metabolic, and profibrotic responses in glomerular and tubular compartments, and has been implicated in podocyte injury and progression of glomerular disease [11, 12]. These signaling pathways are regulated independently of TP activation and do not require C5b-9 formation.

C5a also mediates potent inflammatory effects through C5a receptor 1 (C5aR1). It promotes neutrophil chemotaxis, degranulation, oxidative burst, neutrophil extracellular traps (NET) formation, and expression of tissue-damaging enzymes [2, 6]. In the glomeruli, excessive C5a increases inflammation and may propagate injury via cellular signaling toward fibrosis [12]. Meanwhile, C3a also contributes to local inflammation, mitochondrial dysfunction and oxidative injury in podocytes [12]. 

In addition to C5aR1, C5a signals through C5a receptor 2 (C5aR2), which is expressed in the kidney and is thought to modulate inflammatory responses, although its role in glomerular disease remains incompletely understood [2, 4].

Collectively, complement activation is not merely a bystander but actively drives glomerular injury.

### Complement regulators

The activity of complement regulators is essential to maintain homeostasis and not harm the self. Hence, the human body has evolved a diverse set of complement regulatory proteins that act through distinct mechanisms depending on their location (Table 1). Plasma regulators primarily control complement activation in the fluid phase by limiting convertase assembly, promoting the degradation of activated fragments, and preventing MAC formation. In contrast, membrane-bound regulators – located on cell surfaces – prevent convertase activity, facilitate factor I-mediated cleavage of C3b and C4b, or block terminal complement assembly, safeguarding self-cells from complement-mediated damage [2, 3, 6, 13].

**Table 1:** Complement cascade regulators

Regulator	Location	Pathway(s) regulated	Primary Function
Factor H (CFH)	Plasma	Alternative	Cofactor for factor I-mediated C3b cleavage
Factor H-related protein 1 (CFHR1)	Plasma	Alternative / Terminal	Inhibits C5 cleavage
Factor I (CFI)	Plasma	Alternative	Proteolytic inactivation of C3b and C4b
C1 Inhibitor	Plasma	Classical and Lectin	Regulates C1r/C1s and inhibits MASP1/2
C4 Binding Protein (C4BP)	Plasma	Classical and Lectin	Inactivates C4b
Membrane Cofactor Protein (MCP / CD46)	Cell surface	Alternative, Classical, and Lectin	Cofactor for factor I-mediated C3b cleavage
Decay Accelerator Factor (DAF / CD55)	Cell surface	Classical and Lectin	Accelerates decay of C3/C5 convertases
Protectin (CD59)	Cell surface	Terminal	Inhibits oligomerization of C5b-8 and C9
Complement receptor I (CR1; CD35)	Cell surface	Alternative / Terminal	Cofactor for factor I-mediated C3b cleavage; Decays the acceleration activity of C3 convertase

Complement factor H (CFH) plays a key role as a fluid-phase regulator of the AP: it binds to C3b and competes with CFB, thus preventing assembly of the C3bBb convertase, and acts as a cofactor for complement factor I (CFI)-mediated cleavage of C3b into inactive fragments (iC3b) [2, 6]. CFH also recognizes polyanionic structures such as glycosaminoglycans and sialic acids, allowing for localized protection [2]. 

Complement factor-H related proteins (CFHR) modulate CFH activity. CFHR-1 has been implicated in inhibiting C5 cleavage, preventing the formation of MAC and release of C5a [6]. Loss of CFHR-1 (via gene deletions) is associated with higher levels of antibodies against factor-H in atypical hemolytic uremic syndrome [14]. However, in IgA Nephropathy, evidence indicates that CFHR may affect CFH function [15]. 

Regulators for CP and LP include C1 inhibitor, that neutralizes the C1r/C1s and MASP-1/2, and C4 binding protein (C4BP), which regulates C4b convertase [13]. 

CD59 is an important regulator of the TP by preventing C5b-C8 association, thus blocking MAC formation. Other important surface-bound regulators include: membrane cofactor protein (MCP/CD46), a CFI cofactor; decay-accelerating factor (DAF/CD55), which destabilizes C3 convertase; and complement receptor 1 (CR1/CD35), also a CFI cofactor that possesses decay-accelerating activity for C3 convertase [6, 13]. 

Differential expression of surface complement regulators, particularly CD59, CD46, and CD55 critically influences whether C5b-9 induces sub-lytic signaling or overt injury, contributing to heterogeneous cellular responses within the glomerulus [3, 6, 13]. 

## Spectrum of complement-mediated renal pathology

Complement involvement in glomerular disease can be categorized as primary complementopathies, in which complement dysregulation is the principal driver; and secondary complement activation, where complement amplifies injury initiated by other pathologies. Moreover, complement activation also has a significant role in the development and progression of kidney allograft pathology.

### Primary complement diseases

#### Atypical hemolytic uremic syndrome (aHUS)

The aHUS is a prototypical complement dysregulation syndrome, classically linked to AP defects. Patients may harbor loss-of-function variants (CFH, CFI, CD46), gain-of-function variant (CFB, C3), or present with anti-factor H antibodies (~ 10% of cases) [1, 3, 6, 7, 16]. Around 3% of patients may carry multiple mutations in complement-related genes [3]. 

#### C3 glomerulopathy (C3G)

C3G – comprising dense deposit disease (DDD) and C3 glomerulonephritis – is defined by predominant glomerular C3 deposition in the absence of significant immunoglobulins [17]. Genetic defects in CFH and C3 have been found in several familial cases of C3G. These abnormalities lead to persistent AP activation and glomerular deposition of C3, causing mesangial and subendothelial lesions. Some patients also harbor nephritic factors, such as C3NeF or C4NeF, which are autoantibodies that stabilize conversates, and end up accelerating complement activation. The diagnosis relies on immunofluorescence (IF) showing C3 deposition in the absence of significant immunoglobulin or other complement components. The diagnosis of DDD is established by the presence of characteristic, intensely osmiophilic intramembranous deposits on electron microscopy, which account for the disease’s distinctive dense appearance [17]. 

#### C4 glomerulopathy (C4G)

C4G is a newly described form of complement-mediated glomerulonephritis that includes two subtypes: C4 dense deposit disease and C4 glomerulonephritis. This disease is distinguished by dominant C4d deposition on immunofluorescence, with minimal or absent staining for C1q, C3, and immunoglobulins. This suggests a predominant activation of the LP, although evidence is limited [18]. 

### Secondary complement activation

#### IgA nephropathy (IgAN)

IgA nephropathy (IgAN) is the most common glomerulonephritis worldwide. Its pathophysiology is classically explained by a four-hit model, comprising: (i) mucosal overproduction of galactose-deficient IgA1 (Gd-IgA1); (ii) generation of IgG autoantibodies directed against these aberrantly glycosylated IgA1 molecules; (iii) formation and systemic circulation of immune complexes; and (iv) mesangial deposition of these complexes within the kidney [19]. 

The role of complement in IgAN extends beyond a passive bystander effect and involves both activation and dysregulation. Gd-IgA1 exposes terminal N-acetylgalactosamine residues that can be recognized by mannose-binding lectin (MBL) and ficolins, thereby triggering activation of the lectin pathway through MASP-1 and MASP-2 [4, 20]. In parallel, polymeric and immobilized IgA1-containing immune complexes are capable of activating the alternative pathway, both in the circulation and after mesangial deposition [19]. 

While soluble IgA1 and its Fab fragments may inhibit classical pathway activation by competing with IgG for C1q binding [21], this regulatory effect is lost once immune complexes are deposited in the mesangium. Importantly, accumulating evidence suggests that complement activation in IgAN is insufficient to ensure effective immune complex clearance, instead favoring their persistence and amplifying local inflammation [4, 19]. Thus, dysregulated complement-mediated immune surveillance may act as an additional pathogenic amplifier within the four-hit model, sustaining mesangial injury and disease progression [4, 5, 19]. 

IgA is the main immunofluorescence deposit, with C3 co-deposition correlating with poorer outcomes [22, 28]. Supporting evidence of LP/AP involvement includes: (i) MBL and L-ficolin deposition in some patients; [20] (ii) C4d deposition is seen in ~ 30% of cases [4]; (iii) rare C1q deposition; [4] and (iv) frequent properdin co-deposition (~ 75%) [23]. 

#### Lupus nephritis (LN)

In LN, immune complex deposition activates CP locally, driving glomerular injury. Paradoxically, deficiency in CP components (C1q/C1r/C1s/C2/C4) predisposes to systemic lupus by impairing apoptotic debris clearance and facilitating autoimmune responses [24, 25]. 

Beyond CP, evidence implicates the AP in LN progression: in active disease, plasma levels of Bb and C5b-C correlate, and AP biomarkers correlate with poorer one-year response to immunosuppressive therapy, as well as higher estimated glomerular filtration rate (eGFR) decline [26, 27]. Temporal patterns of complement dynamics suggest that isolated C4 low levels may signal early CP activation, whereas isolated C3 low levels associate with overt flares, implicating C3 amplification via AP [28]. 

Kidney biopsies often show “full house” pattern in immunofluorescence (deposition of immune complexes and C1q, C3, and C4) [1]. 

In addition to immune complex–mediated classical pathway activation, autoantibodies directed against C1q play an important role in lupus nephritis. Anti-C1q antibodies are strongly associated with renal involvement in systemic lupus erythematosus and correlate with disease activity, particularly proliferative forms of lupus nephritis. These antibodies bind to C1q deposited in glomeruli or to C1q bound to immune complexes, promoting sustained classical pathway activation, local complement amplification, and inflammatory injury, and likely contribute to the prominent glomerular C1q staining characteristic of lupus nephritis [24–26]. 

#### Antineutrophil cytoplasmic antibody (ANCA)-associated glomerulonephritis (ANCA-GN)

ANCA-GN is classically pauci-immune, but complement activation plays an increasingly appreciated role in its pathogenesis. ANCA-driven neutrophil activation causes degranulation, reactive oxygen species production, microparticle and NET release, endothelial injury, and activation of the coagulation cascade; these events engage the AP, generating C5a, which in turn amplifies neutrophil recruitment and activation via C5aR1 signaling—a feed-forward inflammatory loop [29]. In vitro, ANCA-exposed neutrophils generate both C3a and C5a, with C5a being key to priming [29]. 

Evidence of AP and TP activation includes: (i) glomerular staining for CFB, properdin, and MAC; [4] (ii) elevated levels of C3a, C5a, C5b-9, Bb in the serum in active disease; [30, 31] (iii) low circulating blood levels of FH [32]. 

#### Anti-glomerular basement membrane (GBM) disease

In anti-GBM disease, autoantibodies bind α3 chain of type IV collagen in the GBM, forming in situ immune complexes that activate CP via C1q binding [4]. MBL deposition has been observed, suggesting involvement of the LP [33], and colocalization of CFB with C5b-9 in crescentic lesions suggests AP engagement [34]. Elevated plasma C5b-9 and urinary C5a correlate with disease severity [35, 36]. 

#### Membranous nephropathy (MN)

Primary MN is strongly linked to antibodies against PLA2R (in ~ 70–80% of cases), as well as other antigens (thrombospondin type I domain-containing 7 A, semaphorin 3B, etc.) [37]. 

Although complement activation is increasingly implicated in MN, the precise pathways and the full extent of its involvement remain incompletely understood.

Evidence includes: glomerular staining for C3, IgG4, FB, and properdin; [38] absence of CR1 in the glomeruli analysed by mass spectrometry [39]; and elevated titers of C5a and higher levels of MAC in the urine [40]. This feature is seems to be shared between all types of MN irrespective of the target antigen, except for PCDH7-associated MN, where C3 deposition is either absent or slightly positive (1+) on IF, a finding confirmed by mass spectrometry [41]. 

In primary MN, the predominant immune response involves IgG4 antibodies, which cannot activate the CP. Hence, it has been widely assumed that this pathway is not central to the pathogenesis of the disease. However, a subset of patients also exhibit IgG1/IgG2/IgG3 subclasses of anti-PLA2R antibodies and C1q deposition, suggesting CP’s role in this disease [42–44]. 

In vitro, galactose-deficient anti–PLA2R IgG can bind MBL, triggering LP activation [45]. Additional support comes from colocalized MBL deposition in capillary walls [46]. 

In passive Heymann nephritis models, sublytic MAC insertion triggers proteinuria [47]. Podocyte upregulation of C3aR has been correlated with worse proteinuria and impaired renal function; blockade of C3aR ameliorates injury in animal models [48]. 

#### Focal segmental glomerulosclerosis (FSGS)

FSGS is a histopathological lesion that can arise from primary, secondary, or genetic causes. Despite often-negative immunofluorescence, IF staining for IgM and C3 is frequently observed in sclerotic portions of the glomerular tufts [49], with IgM thought to initiate local complement activation [50]. 

Elevated plasma and urinary complement activation products from the alternative (Ba, Bb), classical (C4a), and terminal (C5b-9, C5a) pathways have been reported in patients with FSGS and were associated with markers of disease activity such as proteinuria and renal function. However, evidence linking these markers to histological severity remains limited [51]. LP involvement is less clear; [52] some biopsies show C4d positivity in absence of C1q, suggesting a potential, albeit minor, LP role [53]. 

#### Monoclonal gammopathies

Increasingly, monoclonal immunoglobulins have been recognized as drivers of complement dysregulation. In older patients with C3G and monoclonal gammopathy, paraproteins may act as C3NeF or autoantibodies against factor H [54]. Some patients show low serum C3 with preserved C4 and elevated C5b-9, consistent with AP/TP activation [55]. Anti–factor H antibodies may also coexist, further supporting the role of AP dysregulation in disease development [55]. 

#### Diabetic kidney disease (DKD)

Although typically not considered immune-mediated, growing evidence implicates complement in diabetic glomerular injury. Advanced glycation end-products can bind to MBL and activate the LP [56]. Elevated circulating MBL levels have been documented in diabetic patients [57, 58]. 

Co-deposition of C1q in DKD has been observed, as well as correlation with higher proteinuria, raising the possibility that the CP may also contribute to disease progression [59, 60]. 

### Complement activation in kidney allograft

Complement activation plays roles at every stage of kidney transplantation: pre-, post- and during transplant [61]. The degree of complement activity is correlated to adverse outcomes, including delayed graft function (DGF) [62]. 

#### Pre-transplantation

In a clinical study, when compared to living donor, donor brain death was associated with induction of intrarenal C3 expression in pre-implantation kidney biopsies, and higher C3 expression correlated with worse early post-transplant allograft function, supporting a link between brain death–related renal complement activation and early graft dysfunction [63]. Mechanistically, brain death has also been reported to induce systemic complement activation with downstream generation of C5a, accompanied by upregulation of tubular C5aR expression and increased intrarenal inflammatory cytokine signals, suggesting a C5a–C5aR axis that couples systemic activation to local renal inflammation [64]. A different study also demonstrated higher expression of complement genes in deceased donor kidneys, compared with living donor kidneys [65]. 

During organ preservation, proteomic analysis of preservation fluids detected complement proteins, with a negative correlation with recipient eGFR one year post-transplantation [66, 67]. 

#### During transplantation

Ischemia-reperfusion Injury (IRI) is a major risk factor for DGF. Reduced oxygen delivery causes oxidative stress and cytokine production, triggering complement activation [68]. Plasma C5b-9 levels during reperfusion correlate with early and 1-year graft function [69]. 

Animal studies implicate AP/LP activation in IRI, not confirmed in kidney recipients [70, 71]. Nonetheless, in cardiac surgery patients with kidney histological lesions similar to IRI, higher urinary CFB levels were found [72]. Higher pre-transplant ficolin-3 titers correlated with worse graft survival, implicating LP contribution [73]. 

#### Post-transplantation

In antibody-mediated rejection (ABMR), donor-specific antibodies activate CP via C1q, initiating complement-dependent endothelial injury [68]. The complement-fixing capacity of donor-specific antibodies (DSA) has important prognostic implications, as C1q-binding DSA are associated with a significantly increased risk of graft loss, while C3d-binding DSA identify an even higher-risk phenotype reflecting more potent and sustained complement activation [74, 75]. Furthermore, it was found that plasma C4a and Ba levels were significantly elevated in patients with antibody-mediated rejection, suggesting a role for the AP as well [76]. The evidence of involvement of the LP is still inconsistent.

The deposition of C4d (C4b’s breakdown product) in peritubular capillaries remains a diagnostic marker of ABMR, although C4d-negative is now also recognized [77]. 

Thrombotic microangiopathy (TMA) represents a severe histopathological pattern of ABMR [77]. In the presence of DSA, TMA has been traditionally attributed to activation of classical complement pathway [78]. However, emerging evidence indicated that terminal complement pathway activation might not be the primary driver of cytotoxic injury in a substantial proportion of ABMR cases [79]. Nonetheless, studies show that clinically, the presence of ABMR-associated TMA has higher risk of premature graft loss compared with other TMAs [80]. 

## Complement-targeted therapies

Complement-targeted therapies have dramatically expanded over the last decade (Table 2). Their implications for specific diseases are more detailed in the following sections.

**Table 2:** Summary of clinical trials evaluating complement-targeted therapies in kidney disease

Target	Drug	Disease	Trial (Name / Phase / NCT ID)	Primary Endpoint	Key Results / Status
C3	Pegcetacoplan	C3G	VALIANT / phase III / NCT05067127	Variation in proteinuria at 26 weeks	68% ↓ proteinuria; >50% pts <1 g/day
	Pegcetacoplan	C3G (post-tx)	NOBLE / phase III / NCT04572854	% reduction of C3 staining	Less C3 staining; stable eGFR
	Pegcetacoplan	C3G, IgAN, LN, MN	DISCOVERY / phase II / NCT03453619	Safety; proteinuria change	Only C3G cohort showed ↓ proteinuria; terminated earlier
	ARO-C3	C3G, IgAN	phase I/IIa / NCT05083364	Safety; proteinuria change	Mean ↓ 41% in proteinuria; Ongoing
C5	Eculizumab	aHUS	phase II / NCT00844545, NCT00844844	Change in platelet count; TMA event-free status	Effective TMA control and renal recovery
	Ravulizumab	aHUS	phase III / NCT02949128	TMA response	Effective in TMA control
	Ravulizumab	IgAN	SANCTUARY / phase II / NCT04564339	% change in proteinuria	~ 30% ↓ at 26 weeks, ~ 45% ↓ at 50 weeks; slower eGFR decline
	Ravulizumab	LN	SANCTUARY / phase II / NCT04564339	% change in proteinuria	Ongoing; no published results yet
	Crovalimab	aHUS	COMMUTE-a, COMMUTE-p / phase III / NCT04861259, NCT04958265	% complete TMA response	Ongoing
	Cemdisiran	IgAN	phase II / NCT03841448	% change in proteinuria	~ 37% ↓ at 32 weeks, slower eGFR decline
C5aR1	Avacopan	ANCA-GN	ADVOCATE / phase III/ NCT02994927	% remission at 26 weeks	Non-inferior to PDN; superior sustained remission at 52wk
	Avacopan	IgAN	phase II / NCT06676579	Change in proteinuria at 12months	Ongoing
	Avacopan				
Factor B	Iptacopan	IgAN	APPLAUSE-IgAN / phase III / NCT04578834	% change in proteinuria; eGFR slope at 24 months	Ongoing; 38,3%↓ proteinuria at 9months
	Iptacopan	aHUS	APPELHUS / phase III / NCT04889430	% complete TMA response	Ongoing
	Iptacopan	LN	phase II / NCT05268289	% complete renal response at 24 weeks	Ongoing
MASP-2	Narsoplimab	IgAN	phase II / NCT 02682407	safety; % change in proteinuria	SS 1: ↓54–95% at 18 weeks SS 2: ↓61,4% at weeks 31–54
	Narsoplimab	IgAN	ARTEMIS-IgAN / phase III / NCT03608033	% proteinuria at 36 weeks	Ongoing
	Narsoplimab	aHUS	phase III / NCT03205995	% platelet count change	Ongoing
	Narsoplimab	IgAN, LN, MN, C3G	phase II / NCT02682407	% of patients with TAE; % change serum and urine complement	Unknown status; no results reported

ANCA-GN: antineutrophil cytoplasmic antibody (ANCA)-associated glomerulonephritis; aHUS: atypical hemolytic uremic syndrome; C3G: C3 glomerulopathy; eGFR: estimated glomerular filtration rate; IgAN: IgA Nephropathy; LN: lupus nephritis; MN: membranous nephropathy; PDN: prednisolone; post-tx: post-kidney transplant; pts: patients; SS: substudy; TAE: treatment adverse effects; TMA: thrombotic microangiopathy

### C3 inhibition / suppression

#### Pegcetacoplan

Pegcetacoplan is a targeted C3/C3b inhibitor that blocks both upstream activation and downstream amplification. In the phase III VALIANT trial, it achieved ~ 68.1% reduction in proteinuria and over 50% of patients attained < 1 g/day proteinuria, along with stabilization of eGFR in *C3G* (native or post-transplant) [81]. In the NOBLE trial, Pegcetaboplan reduced C3 staining on biopsy, lowered proteinuria by ~ 54%, and increased serum C3 in *post-transplant C3G* [82]. 

In the broader phase II DISCOVERY trial, only the C3G arm yielded meaningful data: >50% reductions in proteinuria at 48 weeks, improvements in serum albumin, and normalization of complement biomarkers such as C3 and C5b-9 [83]. Data remain limited for non–C3G glomerulopathies.

#### ARO-C3

ARO-C3 is an RNA interference therapy designed to suppress hepatic production of C3. In a phase 1/2a clinical trial (NCT05083364) enrolling patients with *IgAN* and *C3G*, interim data demonstrated reduction in spot urinary protein-creatinine ratio of ~ 41%, as well as C3 levels reduction. The trial remains ongoing.

### C5 inhibition / suppression

#### Eculizumab

Eculizumab is a monoclonal antibody that binds C5 and prevents its cleavage into C5a and C5b, thereby halting MAC formation. It requires intravenous infusions every 2 weeks, and it has dramatically improved outcomes in *aHUS* patients [84]. 

In medium and high risk *aHUS transplant recipients*, prophylactic eculizumab has shown improved renal allograft survival at 1 year post-transplant (97% versus 64% in untreated patients) [85]. 

Regarding *aHUS recurrence*, treatment with eculizumab was highly effective as a treatment option [86, 87]. 

The optimal duration of eculizumab in complement-mediated aHUS remains uncertain. Prospective and observational data suggest that elective discontinuation can be reasonable in carefully selected “lower-risk” patients, particularly those without pathogenic complement gene variants once kidney function has stabilized [88]. Patients who have pathogenic complement mutations (CFH/MCP) have a substantially higher risk of relapse [89] and should undergo close monitoring after Eculizumab discontinuance. Importantly, treatment with Eculizumab after relapse is usually effective when restarted earlier [88, 90] The KDIGO consensus recommends that discontinuation be considered mainly in patients without pathogenic variants, while decisions in those with pathogenic variants or persistent high-titer anti–factor H antibodies should be individualized; it urges extreme caution in CKD G3b–G5 and kidney transplant recipients, where longer-term therapy is often favored [5]. 

The consensus recommends monitoring the adequacy of terminal complement blockade during treatment through assessment of total hemolytic complement activity (CH50), with optimal suppression to < 10%, and measurement of Eculizumab trough levels when available. Following treatment discontinuation, close surveillance for early signs of aHUS relapse is advised, including monthly laboratory evaluation – typically comprising kidney function, complete blood count with platelet count, and markers of hemolysis – as well as weekly urinary dipstick testing [5]. 

In *ABMR*, the evidence for Eculizumab is heterogeneous. A trial evaluating Eculizumab for ABMR was reported as negative when compared to plasmapheresis and intravenous immunoglobulin (unpublished; NCT01895127). Nevertheless, several case reports and small series have shown potential benefit of Eculizumab as an adjunct to standard therapies in severe or refractory ABMR [91, 92]. 

The role of Eculizumab in preventing *IRI* has yielded heterogeneous clinical results. In a pediatric kidney transplant study, peri-transplant administration of eculizumab was associated with improved early graft function and reduced histological markers of chronic injury, including arteriolar hyalinosis and chronic glomerulopathy [93]. In contrast, a randomized clinical trial in adult kidney transplant recipients demonstrated no significant improvement in graft function at 6 months among patients treated with eculizumab compared with standard care [94]. These discordant findings suggest that the efficacy of terminal complement blockade may be influenced by recipient age, donor characteristics, timing of administration, and the multifactorial nature of ischemia–reperfusion injury.

#### Ravulizumab

Ravulizumab, a long-acting anti-C5 antibody [95], reduced proteinuria in *IgAN* patients by 30% at 26 weeks and 45% at 50 weeks, with a slower decline in eGFR (–3.9 vs. − 6.3mL/min/1.73 m²) relative to placebo (SANCTUARY trial) [96]. Results from the LN arm of the trial are pending. A phase III trial (NCT06291376) is underway.

In *aHUS kidney transplant recipients*, case reports and small series have described sustained remission without TMA recurrence after kidney transplantation in patients maintained on Ravulizumab [97, 98]. 

#### Crovalimab

Crovalimab is a long-action, subcutaneous anti-C5 monoclonal antibody currently being evaluated for *aHUS* in two phase 3 trials (COMMUTE-a in adults, and COMMUTE-p in pediatric patients). This drug binds to a different epitope than eculizumab/ravulizumab, potentially offering an option for patients with C5 variants (e.g., p.Arg885His) associated with poor response to those agents [99]. 

#### Avacopan

Avacopan is an oral selective C5a receptor inhibitor, designed as a corticosteroid-sparing strategy in *ANCA-GN*. In the phase III ADVOCATE trial, those receiving Avacopan demonstrated a superior recovery of eGFR compared with those receiving Prednisone, both at 26 weeks (5,8 vs. 2,9mL/min) and at 52 weeks (7,3 vs. 4,1mL/min), with greatest gains among those with baseline eGFR < 30 ml/min (13,5mL/min vs. 8,2mL/min) [100]. 

Furthermore, patients allocated to Avacopan reported significant improvements in quality-of-life metrics and experienced a substantially lower incidence of corticosteroid-related complications [100]. 

Hence, 2024 KDIGO glomerular disease guidelines now include Avacopan as a glucocorticoid alternative [101]. 

A pilot study in *IgAN* showed proteinuria improvements, although the number of patients enrolled limited the evidence [102]. There is currently a phase II trial taking place, with no results yet (NCT06676579).

In *C3G*, a phase 2 trial (ACCOLADE) did not meet its primary endpoint consisting of change in histologic disease activity at week 26 versus placebo, suggesting that blocking C5aR1 alone may be insufficient in many patients, and supporting the importance of additional AP effector mechanisms beyond C5a in C3G [103]. 

#### Cemdisiran

Cemdisiran, an RNA interference agent targeting hepatic C5 synthesis, achieved a 37.4% reduction in proteinuria at week 32 and attenuated eGFR decline (–6.76 vs. − 11.9 mL/min/1.73 m² per year) in a phase II trial in *IgAN* [104]. 

### Factor B inhibition

#### Iptacopan

Iptacopan is a small molecule inhibitor of factor B, targeting the AP specifically. In *IgAN*, the phase III APPLAUSE IgAN trial reported a 38.3% proteinuria reduction at 9 months, along with hematuria reduction [105]. 

In the APPEAR-C3G, Iptacopan achieved 35% proteinuria reduction at 6 months vs. placebo, sustained at one year in *C3G* patients, that was sustained at 12 months [106, 107]. 

A phase III trial (APPELHUS; NCT04889430) in *aHUS* patients is currently ongoing. The role in *LN* patients remains unclear, with an ongoing phase II study (NCT05268289).

### MASP-2 inhibitor

#### Narsoplimab

Narsoplimab is a humanized monoclonal antibody against MASP-2, aiming to inhibit LP activation selectively. In a phase II study in *IgAN*, 18-week use led to reductions in proteinuria while preserving eGFR [108]. A phase III trial is ongoing. Narsoplimab is also being explored in refractory *aHUS* (NCT03205995) and in glomerular diseases – *MN*, *LN*, and *C3G* – although data are pending.

## Challenges and future directions

There has been a rapid expansion of complement-targeted therapies for kidney disease. Early trials show antiproteinuric effects and short-term benefits, but long-term efficacy and safety remain unclear. Given the central role of complement in host defense, infectious risk – particularly *Neisseria* infections with terminal pathway blockade – remains a major concern and requires structured risk-mitigation strategies (vaccination and, in selected settings, prophylactic or reserve antibiotics) [4, 109]. Patients undergoing concomitant immunosuppression, including those with SLE or IgAN, may face an elevated risk of infection, underscoring the need for careful evaluation when considering complement-targeted therapy [4]. 

Safety considerations may differ by target depth: proximal inhibition may provide broader disease control but potentially greater interference with protective functions, and treatment interruptions may carry a risk of “breakthrough” complement-mediated events, highlighting the importance of adherence, monitoring and careful target selection [109]. 

Uncertainties persist regarding treatment duration, patient selection based on pathway activation, and the high cost and limited accessibility of these agents, raising cost-effectiveness issues [110]. Future trials should incorporate standardized risk assessment and mitigation strategies, alongside robust biomarker and endpoint frameworks and long-term safety monitoring [110]. 

Regarding diagnosis, laser capture microdissection and mass spectrometry enable precise identification of complement components [111]. Distinct activation patterns have been described – for instance, C4a dominance in MN versus C4b in SLE – suggesting improved diagnostic precision [111]. 

Gene therapy is emerging, with adeno-associated virus delivery of factor H or mini-FH showing durable control of complement dysregulation in preclinical models [112, 113]. 

Combination strategies may further enhance outcomes when complement inhibitors are used alongside standard therapies.

## Conclusions

Significant progress has been made in clarifying the role of complement in various glomerular diseases, yet several mechanisms in this intricate network of pathways remain incompletely understood. Gaining deeper insight into the specific pathways of complement activation will allow for the identification of more precise therapeutic targets.

Recent advances have profoundly reshaped our understanding of the complement system, paving the way for therapeutic innovations that are already transforming clinical practice. In the past years, we have witnessed remarkable progress in finding effective complement-targeted therapies. Complement inhibition, once a theoretical concept, has now become a clinical reality not only for aHUS, but also with approved and emerging agents demonstrating transformative benefits in other diseases, such as C3G, ANCA-GN, and IgAN. These milestones highlight the potential of these drugs not only to control the disease activity, but also to actively modify patient prognosis, which cannot be overlooked.

Despite remaining challenges – including the need for robust long-term data, optimized patient stratification, and clearer identification of those who derive the greatest benefit – the trajectory of research is undoubtedly promising. Complement-targeted therapies represent one of the most exciting frontiers in Nephrology, and moving forward, integrating genetic, proteomic, and biomarker-based stratification will be key to personalized therapy.

## Data Availability

No datasets were generated or analysed during the current study.

## Glossary

**Alternative pathway (AP)**: One of the three complement activation pathways, continuously active at low levels and tightly regulated.
**Anaphylatoxins (C3a, C5a)**: Small complement-derived peptides with potent pro-inflammatory and chemoattractant effects.
**C3 convertase**: Enzyme complex responsible for cleavage of C3 into C3a and C3b, central to amplification of complement activation.
**Classical complement pathway (CP)**: Complement activation pathway triggered by antigen–antibody immune complexes, primarily involving IgG or IgM.
**Lectin pathway (LP)**: Complement activation pathway initiated by pattern recognition molecules binding to microbial carbohydrates.
**Membrane attack complex (MAC, C5b-9)**: Terminal complement complex that forms transmembrane pores and induces cell injury.
**Pauci-immune glomerulonephritis**: Glomerulonephritis with minimal or absent immunoglobulin and complement deposition on immunofluorescence, typical of ANCA-associated vasculitis.
**Primary complement-mediated kidney disease**: Renal disorders in which complement dysregulation is the principal pathogenic driver, such as C3 glomerulopathy.
**Secondary complement-mediated kidney disease**: Renal disorders in which complement activation contributes to disease pathogenesis as a downstream or amplifying mechanism, rather than being the primary driver.
**Terminal pathway (TP)**: The final stage of complement activation, involving cleavage of C5 and assembly of the membrane attack complex (C5b-9).
